# Developing the collection of statistical food waste data on the primary production of fruit and vegetables

**DOI:** 10.1007/s11356-020-09908-5

**Published:** 2020-07-09

**Authors:** Katri Joensuu, Hanna Hartikainen, Sirpa Karppinen, Anna-Kaisa Jaakkonen, Mika Kuoppa-aho

**Affiliations:** 1 Natural Resources Institute Finland (Luke), Maarintie 6, 02150 Espoo, Finland; 2 Natural Resources Institute Finland (Luke), Latokartanonkaari 9, 00790 Helsinki, Finland

**Keywords:** Food loss, Side flow, Agriculture, Horticulture, Potatoes, Carrots, Strawberries, White cabbage

## Abstract

In order to meet global goals of reducing food waste, feasible monitoring methods to verify the impact of reduction measures are needed. In this study, a method was developed for gathering food waste data related to the primary production of fruit and vegetables using a questionnaire for farmers. A data collection form was planned and tested for this purpose. Data was collected on the volumes of different uses of yields and the reasons why part of the yield does not end up in food use. The crop species that were included in this pilot study were food potatoes, carrots, white cabbage, and strawberries. In primary production, the share of food use of the total yield was highest for potatoes (96%) and lowest for carrots (72%). In the case of strawberries and white cabbage, 86% and 90% were used as food, respectively. In the future, it is recommended that this kind of survey be added to annual crop production surveys that cover agricultural and horticultural enterprises in Finland. To improve the response rate and decrease the reporting load of farmers, it is important that the questionnaire is designed to be as easy as possible to answer.

**Electronic supplementary material:**

The online version of this article (10.1007/s11356-020-09908-5) contains supplementary material, which is available to authorized users.

## Introduction

It has been estimated that up to one third of the food produced for human consumption is lost or wasted globally (Gustavsson et al. 2011), meaning that the resources used for this food production are also lost. The United Nations (UN) has set a sustainable development goal that aims to halve global food waste at the retail and consumer levels and reduce food losses in production and supply chains by 2030 (UN 2016). This goal is also supported by the European Commission (EU 2015). According to the Commission’s ‘resource efficient Europe’ roadmap, the aim is to reduce waste generation and utilize all waste as a resource by 2020 (European Commission 2011). To meet these targets, the European Commission has established a common enactment for its member states to monitor food losses and waste in all steps of the food chain (European Commission 2019). Food losses occur at the first stages of the food supply chain: primary production and food industry, whereas losses occurring at the retail and consumption stages are referred to as food waste (Parfitt et al. 2010).

The European Commission target of monitoring food waste creates pressure to improve food waste monitoring in the member states. In industrialized countries, food is mainly wasted at the consumption level (Gustavsson et al. 2011), and hence, the focus needs to be at this level. However, consumers can also cause food waste indirectly, because supply chain actors, such as retailers, assume that consumers demand perfect cosmetic quality related to the size, shape, and appearance of food products (Göbel et al. 2015, de Hooge et al. 2018), which leads to losses and waste in the primary production stage of the food chain. In fact, in primary production, especially fruit, vegetables, roots, and tubers have been shown to suffer from relatively large losses varying from 10 to 30% of the production volume (Gustavsson et al. 2011; Hartikainen et al. 2018). Food loss and waste in the primary production of potatoes, vegetables, and fruit have been quantified in several studies in several European countries, namely, the UK: Beausang et al. (2017) and Terry et al. (2011); Switzerland: Beretta et al. (2013); Sweden: Davis et al. 2011, Jordbruksverket (2009), Olsson et al. (2011), and Strid et al. (2014); Finland: Hartikainen et al. (2014); the Nordic countries: Hartikainen et al. (2017); France: Redlingshöfer et al. (2017); Belgium: Roels et al. (2010); Germany and Austria: Schneider et al. (2019), as well as in the USA (Johnson et al. 2018) and Australia (McKenzie et al. 2017). Porter et al. (2018) have recently reviewed European studies related to the food losses in the production of fresh fruit and vegetables caused by cosmetic faults, defects, and blemishes. Other than quality requirements related to the cosmetic characteristics of the products, especially overproduction and difficulties to find suitable buyer for the whole yield, as well as disadvantageous weather conditions, pests, and plant diseases, are important reasons for on-farm losses.

It should also be noted that products with cosmetic faults, defects, and blemishes could also be further processed to produce other food products (Mattsson 2014). There are even companies whose whole business idea is based on the utilization of these kinds of fruits and vegetables (Joensuu 2018).

While food waste has been studied extensively in recent years (Møller et al. 2014; Stenmarck et al. 2016), the data on waste is still often limited to small and/or skewed samples (Hartikainen et al. 2018; Xue et al. 2017). Most of the previous assessments on food losses and waste in primary production are based on expert estimates or interviews with only a few farmers, and it has been stated that there is still a great need for more detailed, systematic, and consistent data about food waste particularly from primary production (Chaboud 2017; Redlingshöfer et al. 2017). For example, there is a risk of underreporting if only questionnaires or interviews for farmers are used in the assessment of food losses and waste in primary production (Johnson et al. 2018). However, field measurements are more labor-intensive than questionnaires and fewer farms can be included, which leads to the sample’s lower representativeness.

The aim of this pilot project was to contribute to the European Commission target to monitor food waste in primary production, with the focus on horticultural products. As a result, a method was established for the collection of food waste data from horticultural producers using a questionnaire, for the purposes of national compilation of statistics on food waste, based on previous work by Hartikainen et al. (2014, 2017). As different food waste definitions exist, the questionnaire was formed so that the data can be adapted for different kinds of reporting purposes using varying food waste definitions. The project was carried out in cooperation between the statistics production and research units of Natural Resources Institute Finland (Luke). The project was carried out between 9 September 2017 and 8 May 2018.

## Materials and methods

In Finland, national waste statistics do not include waste from agriculture (Statistics Finland 2018). The current Finnish waste statistics only monitor municipal waste, which includes all types of waste from the food chain, and there is no common system for monitoring the actual amount of food going to waste in Finland. Hence, there is no monitoring system for providing comprehensive knowledge on the amounts and causes of losses and waste throughout the supply chain. Due to the lack of such a monitoring system, food waste and side flows in agriculture have been previously studied in two projects in Finland (Hartikainen et al. 2014, 2017). In the fruit, vegetable, and tuber category, these studies included the production of iceberg lettuce, strawberries, potatoes (Hartikainen et al. 2014), carrots, onions, and green peas (Hartikainen et al. 2017). The studies were based on questionnaires sent to farmers, representing 6–25% of the annual production of the studied crops.

In the current study, information from the past studies was used, and a questionnaire (Appendix 1) was designed to collect data from horticultural producers. The most important fruit, vegetable, and tuber crops in terms of total yield and production value were selected for this study. From open-field vegetables, carrots and white cabbage were selected, potatoes were selected from tuber crops, and strawberries from the fruit and berry production category.

To allow direct comparison with the previous studies (Hartikainen et al. 2014, 2017), the background information (e.g., response rates) are presented and results of the previous studies are attached to the data from the present study in the sections concerning data subjects (“Data subjects”) and results (“Results”). The differences between the studies are further discussed in “Differences compared to previous national studies”.

### Definitions and terminology

Currently, several definitions for food waste are used. Some definitions include only the parts intended for food use (Hartikainen et al. 2017), while others also consider non-food parts such as peel as food waste (Östergren et al. 2014). To solve this problem, the EU Commission has instructed a task group to come up with a uniform definition of food waste, which will be used in the monitoring of food waste in member states.

**Fig. 1:**
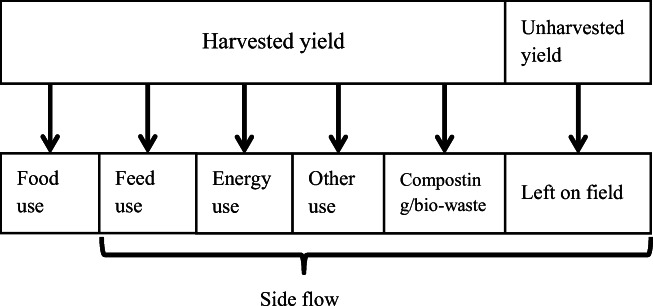
Uses of yield and terminology in this project

The term “food waste” is not commonly used in horticultural production in Finland and may lead to variation in the answers due to the various ways of understanding the term. According to the previous studies (Hartikainen et al. 2014, 2017), the term “waste” in the Finnish language is mostly used to refer to materials that are handled as municipal waste, rather than horticultural products that are, for instance, composted on the farm. The term “loss” is commonly perceived to mean a storage loss, which is only a part of the food waste and loss that needs to be considered. As the terms food waste and “food loss” are not clear, to avoid misinterpretations and ambiguity, it was decided to ask the respondents where their yield ends up (food use, feed use, composting/bio-waste, energy use, left in field, or for some other use (Fig. 1)), and why part of the yield is not used for food. This allows the results to be used to calculate the amount of food waste using different food waste definitions. When needed, the term “side flow” was used to describe biomass that is initially produced for food use, but for some reason is used for something else or is not used at all (left on field) (Fig. 1).

### Questionnaire

In the following, the content of the questionnaire and the answer options (Table 1) are described in detail. The use of crops includes all activities on the farm (storage, post-harvest treatment, packaging) before any further processing (1). Farmers were also asked to define a percentage-based distribution of the applications for which their crops are used (1). In addition, farmers were asked to describe why part of their harvested and unharvested crop is used for purposes other than food (2 and 3). Unharvested crops only included ready-to-be-harvested crops. Farmers were asked to indicate the volume of unharvested crops (2 and 3). The whole questionnaire is presented in Appendix 1.

**Table 1:** Answer options

1. Uses of the harvested yield, divided by volume	- Use as food, including any further processing on the farm - Animal feed - Composting/bio-waste collection - Energy - Other, please specify (open-ended answer).
2. Reasons for unharvested crops, divided by volume	- Factor related to size, weight, shape or appearance / the buyer’s criteria not fulfilled - Larger crops than expected / crops ready for harvesting earlier than expected - Difficulties with sales - Storage losses, rotten - Other, please specify (open-ended answer)
3. Reasons for harvested crops NOT being used as food, divided by volume	- Overproduction/not profitable/no suitable buyer - Factor related to size, weight, shape or appearance / the buyer’s criteria not fulfilled - Availability of workforce - Technical problems (e.g., damaged lifting equipment) - Harvesting losses (e.g., some crops pass through lifting equipment or not all strawberries are picked) - Weather - Diseases, pests - Other, please specify (open-ended answer)

The content of the questionnaire was discussed at several project meetings between different experts who represented Luke’s official statistics (crop and horticultural statistics) and Luke’s food waste research team. The concepts used on the drafted questionnaire were tested by means of a group interview conducted by the Questionnaire Testing and Design team at Statistics Finland (Statistics Finland 2017). The testers were recruited from Luke (many Luke employees are part-time farmers). The requirement was that each tester must be a farmer or otherwise involved in horticultural production. As a result, the group consisted of eight people as requested by Statistics Finland.

With regard to the data system required for the collection of data, the project team cooperated with the Centre for ICT Services of the National Land Survey of Finland which maintains Luke’s data collection programs. The tested questions were entered into the data system, and the verifiers required, and instructions on how to complete the form were also added. The name and contact details of each respondent were auto-filled, together with the crop-specific yield in 2017 in kilos (potatoes/carrots/cabbages/strawberries). The respondents were unable to change these details but were able to enter additional information in the comment field. The auto-filling was carried out in accordance with the crop production survey (on potatoes) or the horticultural production survey (on carrots, cabbages and strawberries) of autumn 2017. Only one crop per farm was included in order to make the questionnaire simpler to understand and faster to fill in. The respondents logged in to the application using a username and password sent to them.

### Data subjects

The data subjects of the project were farms that produce horticultural products and/or potatoes. All the data subjects were selected from among those who responded to the production surveys in autumn 2017 in order to enable the auto-filling of yield values. Producers of carrots, cabbages, and strawberries were selected from the horticultural production survey, and producers of potatoes were selected from the crop production survey.

Greenhouse producers were excluded from the survey as their inclusion would have required a separate data collection system for reasons of data protection. Only Finnish-speaking farms were included, as translating the questionnaire and the terms into Swedish (the other official language in Finland) would have required more specific expertise and testing time. With regard to farms producing several of the studied crops, only the crop with the largest surface area was selected. The study ensured that the pilot study conducted for farms produced a sufficient amount of data for further purposes.

The farms (578 farms) were distributed as follows (proportion of full-country yield in brackets): 133 potato farms (77%), 37 cabbage farms (74%), 58 carrot farms (91%), and 350 strawberry farms (91%). The average response rate of acceptable answers varied between 23 and 38% (Table 2). The data was collected from 19 February to 13 March 2018.

**Table 2:** Response rates in primary production the present study compared to those of Hartikainen et al. (2014, 2017)

Product	Response rate, % (accepted responses)	Number of accepted responses/questionnaires sent	Share of final total yield of the crop, %	Reference
Carrots	25	14/58	14	The present study
	10	27/293	7–8	Hartikainen et al. (2017)
Food potatoes	23	31/133	10	The present study
	14	72/497*	6	Hartikainen et al. (2014)
White cabbage	38	14/37	18	The present study
Strawberries	34	116/350	21	The present study
	21	68/317	13	Hartikainen et al. (2014)

*The questionnaire was sent to all potato producers but only farmers with early potatoes and engaged in food potato production were included in the assessment (excluding feed, starch and seed potato production)

## Results

By combining the data on the uses of harvested yield and the share of unharvested yield, the total side flows not used for food were calculated. The share of food use of the total yield was highest for potatoes (96%) and lowest for carrots (72%) (Table 3). In the case of strawberries and white cabbage, 86% and 90% were used as food, respectively. For feed use and composting, the shares were largest for carrots, 11% and 5%, respectively. The share of the unharvested yield was largest for strawberries (12%) and carrots (11%). Variation in the total shares of side flow was relatively large for all of the studied crops except white cabbage (Table 4). Variation was largest for strawberries, 0–100%, followed by carrots, 0–79%, and food potatoes, 0–40%, depending on the farm. For white cabbage, the share of side flow varied from 0 to 10%. The standard deviation of the share of side flow was 4% for white cabbage and more than 10% for the other crops.

**Table 3:** The uses of yields in primary production; data from previous studies by Hartikainen et al. (2014, 2018). Comparing data from the questionnaires sent in Finland

Product	Food use	Left in field	Feed use	Composting/bio-waste	Energy use	Other	Reference
Carrots	72%	11%	11%	5%	0%	2%	The present study
	74%	4%	11%	8%	0%	3%	Hartikainen et al. (2017)
Food potatoes	96%	1%	1%	1%	0%	1%	The present study
	89%	2%	3%	1%	0%	5%	Hartikainen et al. (2014)
White cabbage	90%	7%	3%	0%	0%	0%	The present study
Strawberries	86%	12%	0%	1%	0%	1%	The present study
	86%	11%	0%	3%	0%	0%	Hartikainen et al. (2014)

**Table 4:** Variation in the results, total side flow in primary production

Product	Weighted mean	Mean	Standard deviation	Variation	Reference
Carrots	28%	30%	23%	0–79%	The present study
	26%	21%	15%	0–50%	Hartikainen et al. (2017)
Food potatoes	4%	7%	12%	0–40%	The present study
	11%	13%	11%	0–50%	Hartikainen et al. (2014)
White cabbage	10%	3%	4%	0–10%	The present study
Strawberries	14%	13%	17%	0–100%	The present study
	14%	17%	14%	2–40%	Hartikainen et al. (2014)

**Fig. 2:**
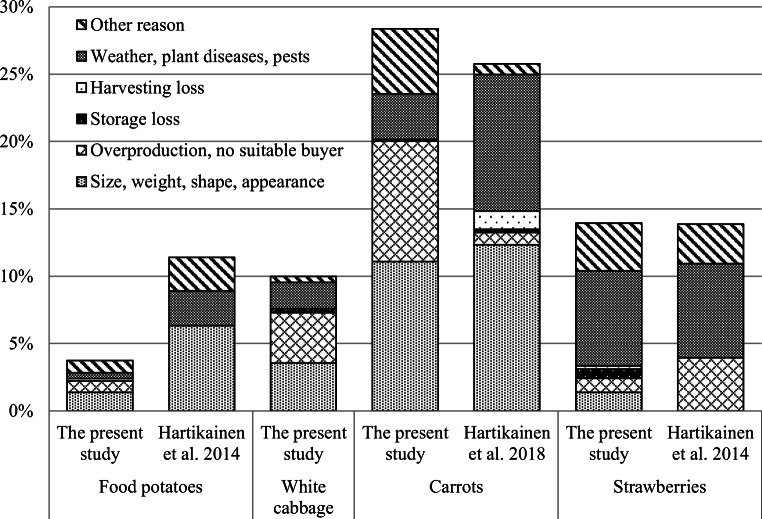
Reasons, why part of the yield is not used as food in primary production; results from the present study compared with data from previous studies by Hartikainen et al. (2014, 2018)

Side flows were also classified according to the reasons why the produce was not used as food. The most important reasons for side flows of potatoes, white cabbage, and carrots were differences in size, weight, shape, and appearance, as well as overproduction (Fig. 2). For strawberries, the most important reasons were weather conditions and plant diseases.

## Discussion

Our results are in the same range as previously reported results for the studied crops in international studies. The on-farm losses have been estimated to be 28% (Davis et al. 2011) and 30–50% (Beausang et al. 2017) for carrots; 27–45% (Terry et al. 2011), 15% (Redlingshöfer et al. 2017), and 1–9% (Schneider et al. 2019) for potatoes; 8% (Davis et al. 2011) and 15% (Johnson et al. 2018) for white cabbage; and 2–10% (Roels et al. 2010), 0% (Davis et al. 2011), 6–8% (Terry et al. 2011), and 1–15% (Beausang et al. 2017). The methodological decisions, namely, choice of food waste definition and assessment methods, are discussed in the next sections (“Shares of food waste according to different food waste definitions” and “Comparison of assessment methods for food waste monitoring”). In “Differences compared to previous national studies”. the results are compared with previous national studies, and in “Differences compared to previous national studies,”the choice of crop species in national food waste monitoring is discussed.

### Shares of food waste according to different food waste definitions

**Fig. 3:**
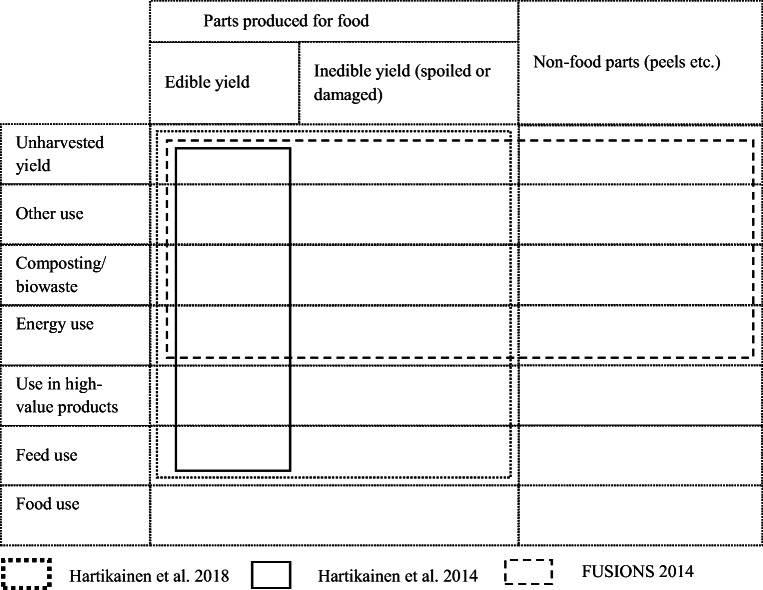
Definition of food waste according to the different food waste definitions

To demonstrate the impact of using different food waste definitions, the share of food waste was calculated for carrots, white cabbage, food potatoes, and strawberries according to three different food waste definitions (Fig. 3; Table 5). As can be seen, the share of the side flows can vary greatly depending on the definition. The definition by Hartikainen et al. (2014) results in the smallest shares. This suggests that the differences in the definitions should be taken into account carefully in the planning of future food reduction targets. According to Hartikainen et al. (2014), the targets should focus on the yield that could still be used as food. Hence, food damaged and spoiled on the farm should not be counted as food waste, because the damage, especially weather damage, is often very difficult or even impossible to avoid. In the latter stages of the production and consumption chain, damaged and spoiled produce is counted as food waste because the damage could be avoided with more careful planning. As different food waste definitions exist, future questionnaires are recommended to be formulated in such a way that the data can be applied using the various definitions.

**Table 5:** Share of food waste in the primary production of carrots, white cabbage, food potatoes, and strawberries according to the different definitions of food waste

Food waste definition	Carrots	White cabbage	Food potatoes	Strawberries
Hartikainen et al. (2018)	28%	10%	4%	14%
FUSIONS (2014)	18%	7%	3%	14%
Hartikainen et al. (2014)	11%	5%	1%	2%

### Comparison of assessment methods for food waste monitoring

To meet the food waste reduction targets set by the UN (2016) and European Union (EU) (2015), the European Commission is about to establish a common enactment for its member states to monitor food waste in all steps of the food chain (EU 2019) at the national level. To do this, an assessment method that is systematic and consistent and relatively low in cost to implement is needed. In the present study, food waste data gathering by questionnaire as part of the annual national statistical crop production surveys has been tested. As also the yield data for national agricultural and horticultural statistics is collected using farmer surveys, it is reasonable to use a similar method also for food waste monitoring.

Farmer questionnaires were preferred in the present study due to their relatively low cost of implementation, thus allowing a relatively large share of farms to be included. The share of side flows in the primary production of potatoes, vegetables, and fruit has previously been assessed using farmer questionnaires in a few research projects (Berkenkamp and Nennich 2015, Beretta et al. 2013, Hartikainen et al. 2014, 2018, Milepost consulting 2012, Snow and Dean 2016). In addition, Beausang et al. (2017), Olsson et al. (2011), Strid et al. (2014), and Terry et al. (2011) have interviewed farmers.

However, there is a risk that the respondents understand the questions differently than the person who formulated them. In the present study, for example, there was an option “Other, please specify” for the question “How and where are harvested crops used?”, and it was selected in some cases where crops were clearly used for food, such as for direct sales, outdoor market sales, and pick-your-own strawberries. In addition, waste and crops that were left in the field were also reported under Other, please specify. This problem can be solved if the data collection can be supplemented with interviews, as the interviewer can give additional information to the respondent if there is a lack of clarity. The risk of misinterpretation can also be reduced by testing the questionnaire beforehand and by giving more detailed crop-specific response instructions.

It can also be difficult for the farmers to estimate the share of the unharvested yield. In previous research projects, the share of the unharvested yield has been estimated based on field measurements for several crop species (Hartikainen et al. 2017; Strid et al. 2014; Johnson et al. 2018; McKenzie et al. 2017). In the study by Johnson et al. (2018) in the USA, field measurements gave remarkably larger estimates for unharvested yields for certain crops (up to 85% of marketed yield for watermelons and 68% for cucumbers) than previous farmer estimates from the same country (e.g. 5% for all vegetables in general, Snow and Dean 2016). They state that there is a risk of underreporting if only farmer questionnaires or interviews are used. However, these results are based on studies conducted in different parts of the country and do not necessarily include the same crops. In a previous study conducted in the Nordic countries by Hartikainen et al. (2017), the share of the unharvested yield of carrots was estimated both by field measurements and farmer questionnaires in Finland and Norway. The results differed relatively little between the two methods (although the measurements were made only on a few farms that were not representative of average carrot farms): the measured unharvested carrot yield was on average 6.2% of total yield in Finland and 4.7% in Norway, while the farmer estimates were on average 4.4% in Finland and 4.5% in Norway. In the same study, similar methods were used to estimate unharvested onion yields in Sweden, and also here, the difference was relatively small 2% vs. 4% for field measurements and farmer estimates, respectively. Furthermore, Strid et al. (2014) studied iceberg lettuce side flows in Sweden using both field measurements and farmer interviews. The share of the total unharvested biomass (also including the lower leaves that are not considered part of the yield) was on average 59% (range 45–67%) according to measurements and 30–50% according to farmer estimates).

It should be noted that field measurements are more labor-intensive than questionnaires and fewer farms can be included. The share of side flows can vary greatly between farms (e.g., between 0 and 50% on carrot farms in Hartikainen et al. 2017), and it is critical to include a representative sample of farms in the data collection. Field measurements are also temporally more limited than questionnaires and interviews because the fields need to be examined as soon as possible after harvests, as wild animals may also visit the fields after harvest and eat part of the yield that is left. Later on, it would also be harder to figure out which part of the yield was still edible during harvest. Another limitation of field measurements is that they only take into account the unharvested yield and do not consider the other losses occurring on the farm, e.g., during storage and sorting. According to our results (Table 4), the unharvested yield may represent only a small share of total side flow depending on the crop species.

Also, the yield data for national agricultural and horticultural statistics is collected using surveys, not direct measurements, leading to similar risk of underestimation. It is justifiable that a consistent method is used for both needs. However, direct measurements could also be applied for selected crops and a more limited number of farms at longer time intervals for benchmarking.

### Differences compared to previous national studies

In the present study, larger response rates and a larger share of the total primary production volumes for Finnish carrots, food potatoes, and strawberries were achieved than in Hartikainen et al. (2014, 2017) (Table 2). However, in the present study, our respondents generally represent larger producers of potatoes and carrots as the total number of respondents was relatively small. For instance, the carrot farms included in the study had around 3 times larger carrot yields (yields over 600 thousand kilos per farm per year) than an average Finnish carrot yield (around 200 thousand kilos per farm per year) (Luke 2017), whereas the average yield size in Hartikainen et al. (2018) was close to the country average (213 thousand kilos per farm per year). Hence, while the present study represents a larger share of the Finnish yield, it over-represents the big farms. This can be a problem when estimating overall waste figures for total Finnish crop production, and hence, it is recommended that all farm sizes are included in future questionnaires.

One possible factor that could have affected the response rates of the questionnaires is their timing. Generally, response rates can be expected to be higher when they are timed so that the farmers are not busy with critical field work operations, such as sowing and planting in the spring or harvesting in the autumn. However, the timing of the questionnaires in the different studies was relatively close to each other in the spring when the growing season has not yet started in Finland. In the study of Hartikainen et al. (2014), the data was collected in March 2013 and in the study of Hartikainen et al. (2017) in April 2014.

The values obtained in the present study on the uses of yields in primary production are relatively similar for carrots and strawberries as in Hartikainen et al. (2014, 2017), but the share of food use of the potato yield seems to be greater in the present study (Table 4). The over-presentation of larger farms could partly explain the difference. Additionally, possible reasons for this difference could be the different weather conditions or market conditions between the years studied.

Variability and standard deviations were relatively large between the respondents both in the present study and the previous Finnish studies (Table 4).

The level of importance indicated for the reasons that part of the yield was not used as food in primary production differ between the studies. The most important reason given for carrots and food potatoes were differences related to size, weight, shape, and appearance, and for strawberries, problems related to weather, plant diseases, and pests in both the present study and Hartikainen et al. (2014, 2018). However, in the present study, the second most important reason given for food potatoes and carrots was overproduction (or difficulties in finding a suitable buyer), which was only of minor importance in Hartikainen et al. (2014, 2017). For strawberries, the second most important reason was differences related to size, weight, shape, and appearance in the present study, and overproduction in Hartikainen et al. (2014).

Without the size limitation, the representativeness of the sample would probably have been much better than the previous studies by Hartikainen et al. (2014, 2017). This is probably because the questionnaire used in the present study is much shorter. Moreover, the Statistical Services unit of Luke is a well-known actor concerning farmer questionnaires, and the researchers of Hartikainen et al. (2014, 2017) did not have this type of credibility to engage farmers to reply to the questionnaires. Hence, it is suggested that the Statistical Services unit of Luke will send out the questionnaires in the future as part of annual crop production surveys that cover agricultural and horticultural enterprises in Finland. The present study was conducted as a separate data collection process in spring, when more information about the use of crops was available. However, since the annual surveys are conducted in the autumn (Luke 2017), respondents will need to estimate the future use of their crops. This will increase the complexity in responding to the survey, and therefore, the collection of data on waste from agriculture should be repeated with less frequency, for example, every 4 years. Telephone interviews are also needed for future questionnaires to improve the response rates.

### Choice of crop species

Fruit and vegetables include a wide range of different crop plants and, as can be seen from our results, the shares of the side flows, as well as the reason that part of the yield is not used for food can vary greatly. However, to avoid excessively increasing the reporting load on farmers, it should be considered whether only a few crop species could be selected to represent larger groups. In previous studies, Hartikainen et al. (2014, 2017) selected plant species that represented a large share of the total national fruit and vegetable production (carrots and potatoes), combined by species in which the part that is harvested as the yield is distinctly different (iceberg lettuce and green peas), although their production volumes are less extensive on the national scale. Also, to account for fruit and berry production, strawberries were included (Hartikainen et al. 2014).

In the future, larger crop groups could be selected, such as greenhouse vegetables, field vegetables, fruit, and berries. For data collection, one or two important crop species from each group could be selected. In Finland, these could be greenhouse vegetables (tomatoes or cucumbers), field vegetables (carrots and white cabbage or onions), fruit (apples), and berries (strawberries). Previously, greenhouse vegetable crops have been excluded from the studies because the share of side flows in their production is very small (Franke et al. 2016). However, the production volume of greenhouse vegetable crops is relatively large in Finland (Luke 2017), and to get an overall picture of fruit and vegetable production, it should also be taken into account.

## Conclusions and recommendations for further steps

The aim of this pilot study was to test a method for the collection of statistical food waste data from horticultural producers which would be suitable for the compilation of national statistics on food waste in primary production to contribute to the EU Commission’s target (2019). The results show that a considerable share of the yield may not end up in food use from primary production due to several different reasons. Our results are in the same range as previously reported results for the studied crops in national and international studies. However, our results show that the share of side flow can vary between 0 and 100% between different farms, so the sample size needs to be relatively large and the farms used in the survey should be selected in such a manner that different size classes and geographical regions are equally represented.

It would be recommendable to carry out food waste monitoring as part of the annual national crop production surveys that cover enterprises producing agricultural and horticultural crops. Questionnaires are preferable to field measurements due to their lower cost, which allows a larger share of farms to be included. It is acknowledged that there is a risk of underestimation when using data gathered by surveys. However, questionnaires are a significantly more cost-effective method in comparison to other methods such as field measurements.

When collecting data from farmers, it is important to design the questionnaire to be as easy as possible to answer: this improves the response rate and reduces the reporting load for the farmers. To avoid misinterpretations, the questionnaires need to be designed carefully, and it is necessary to give more detailed crop-specific instructions to the respondents. It is also important to ensure the representativeness of the results by including different types of farms according to the national distribution of farm sizes. To ensure that the data can be applied when using different food waste definitions, it is recommendable to include a sufficient number of questions in future questionnaires.

## Electronic supplementary material

ESM 1 (DOCX 16 kb)
