# Supplementary material for: Developing the collection of statistical food waste data on the primary production of fruit and vegetables
Source: Environ Sci Pollut Res Int. 2020 Jul 9;28(19):24618–27. doi: 10.1007/s11356-020-09908-5 (PMC8144158; doi:10.1007/s11356-020-09908-5)
Supplement: Supplementary file 1 — (DOCX 16 kb) [file 11356_2020_9908_MOESM1_ESM.docx]

**Appendix 1**. Survey questions

| **Survey on the use of carrots, white cabbages, food potatoes and strawberries** |
| --- |
|  |
| **1.**  **Use of crops on the farm** |
|  |
| **According to the crop and horticultural production survey, your farm produced the following total yield in (previous year):**  Instructions: Auto-filling was carried out in accordance with the crop production survey (for potatoes) or the horticultural production survey (for carrots, cabbages and strawberries). Only one crop per farm was included.  ……………… (name of crop) ____ kg *(auto-filled data; cannot be changed by respondents)* |
| **How and where are harvested crops used?**  Instructions: If detailed data about volumes is not available, please estimate on the basis of the average volume for three previous years. This includes all activities on the farm (storage, post-harvest treatment, packaging) before any further processing.  ___ % used for food, including further processing  ___ % used as animal feed  ___ % used for composting/bio-waste collection  ___ % used for energy production  ___ % other, please specify____________________  *(A sum calculator to be added, together with a verifier that the total sum is 100% 🡪 a message will provide a prompt if the sum is lower)* |
| **What are the reasons that part of the harvested crops are used for purposes other than food? Select 1-2 key reasons:**  (Verifier: If 100% are used for food in the previous question, this question can be ignored.)   \| Factor related to size, weight, shape or appearance / the buyer’s criteria not fulfilled \|  \| \| --- \| --- \| \| Larger crops than expected / crops ready for harvesting earlier than expected \|  \| \| Difficulties with sales \|  \| \| Storage losses, rot \|  \| \| Other, please specify \|  \| |
|  |
| **2.**  **Volume of unharvested crops** |
|  |
| **Estimate the volume of unharvested crops.**  Instructions: If there are no unharvested crops, enter zero.  ……………… (name of crop)____ kg |
| **Why did crops remain unharvested**? Select 1–2 key reasons: *(If the previous answer was zero, this question will not be shown.)*   \| Overproduction/not profitable/no suitable buyer \|  \| \| --- \| --- \| \| Factor related to size, weight, shape or appearance / the buyer’s criteria not fulfilled \|  \| \| Availability of workforce \|  \| \| Technical problems (e.g. damaged lifting equipment) \|  \| \| Harvesting losses (e.g. some crops pass through lifting equipment or not all strawberries are picked) \|  \| \| Weather \|  \| \| Diseases, pests \|  \| \| Other, please specify \|  \| |
|  |
